# Narrative abilities of autistic and non‐autistic adolescents: The role of mentalising and executive function

**DOI:** 10.1002/aur.3272

**Published:** 2024-11-19

**Authors:** Anna Harvey, Helen Spicer‐Cain, Nicola Botting, Lucy Henry

**Affiliations:** ^1^ Department of Language and Communication Science, City St George's University of London Northampton Square UK

**Keywords:** adolescents, executive functioning, language, social cognition & Theory of Mind

## Abstract

Spoken narrative skills are important for adolescents in their everyday lives. Previous research suggests that producing well‐structured and coherent narratives may be challenging for autistic young people. Mentalising, also known as “advanced Theory of Mind” (ToM) and “Executive Function” (EF) are two cognitive abilities frequently explored in relation to autism, both of which may be implicated in narrative ability. The present study investigated these relationships in a group of autistic adolescents (*N* = 44) aged 11–15 years and a comparable non‐autistic group (*N* = 54) that did not significantly differ on age, sex, nonverbal cognitive ability, or receptive/expressive language skills. Participants were assessed on a video‐based spoken narrative task, scored for both overall structure (“story grammar”) and narrative coherence. A battery of tasks measuring mentalising and EF (working memory, inhibition, shifting, generativity) was also administered. Relationships between scores on cognitive measures and narrative performance were investigated using hierarchical linear regression analyses. Mentalising scores were found to significantly predict narrative performance across all outcome measures and were a stronger predictor than diagnostic group. Diagnostic group predicted narrative structure (“story grammar”) scores but not coherence scores. EF scores were not predictive of narrative ability in this sample. Mentalising skills appear to play an important role for both autistic and non‐autistic adolescents in the generation of narrative structure and coherence within spoken accounts.

## Lay Summary

Previous research suggests that autistic young people often experience challenges with spoken narrative skills, and that this may be linked to cognitive differences in “mentalising” (inferring what others are thinking or feeling) and high‐level thinking skills executive function, (EF). This study investigated how mentalising and EF contributed to the structure and coherence of narratives produced by a large sample of autistic and non‐autistic adolescents with similar cognitive and language levels. Mentalising ability consistently predicted both narrative structure and coherence, whether participants were autistic or not, but EF did not predict either aspect of narrative performance in this sample.

## BACKGROUND

Narrative, or storytelling, is a sophisticated form of discourse drawing on a range of linguistic, cognitive, and pragmatic abilities (Norbury et al., 2014). Narrative skills are fundamental to human communication, social development and learning, and are essential for both social and academic success (Petersen et al., 2008). Autism is a neurodevelopmental difference with an estimated prevalence of 1.57% amongst school‐aged children in the UK (Baron‐Cohen et al., 2009). Autism is known to impact on young people's communication abilities (American Psychiatric Association, 2013), particularly in terms of pragmatics, that is, the effective use of language in social contexts (Naigles & Chin, 2015). Previous research into the narrative skills of autistic children and adolescents has resulted in conflicting findings across various experimental tasks (Baixauli et al., 2016), with differences in the group matching strategies used by research groups further complicating the picture. However, there is evidence to suggest that autistic young people tend to find generating well‐structured and coherent verbal accounts more challenging than non‐autistic young people (Baixauli et al., 2016; Conlon et al., 2019; Hewitt, 2019).

Structure and coherence are two closely related, yet conceptually distinct, aspects of narrative production (Harvey et al., 2023). The overall structure (macrostructure) of a narrative account reflects how the content of the story is organized. “story grammar” (Stein & Glenn, 1979) is a common approach to macrostructural analysis that considers how narratives are structured around key story elements, such as setting, initiating event, plan, action, consequence, internal response, and resolution. Whilst coherent narratives must have a logical story structure, the concept of narrative coherence was also considered in the present study because it encompasses the broader ways in which narrators create holistic and meaningful accounts. For example, using accurate reference chains when describing the actions of individual characters, so that the listener can easily follow the story, or leaving out irrelevant or incongruous information, which could confuse the audience (Harvey et al., 2023).

Spoken narrative abilities are important for adolescents across many different contexts; for example, sharing experiences with their peers, demonstrating their learning in the classroom, or telling someone about their day (Petersen et al., 2014). To better support the functional communication skills of autistic young people, it is of interest to consider some of the underlying cognitive factors that may be implicated in the production of well‐structured and coherent narratives. Mentalising, also known as “advanced Theory of Mind” (ToM) and Executive Function (EF) are two cognitive abilities that have been extensively researched in relation to autism (Rajendran & Mitchell, 2007), although the relationship between these constructs remains unclear (Hill, 2004). Mentalising refers to the cognitive ability “to attribute mental states to another person and to infer their underlying intentions, thoughts, emotions, and motivation” (Colle et al., 2008, p. 28). The term “advanced ToM” is also sometimes used to distinguish these more nuanced “mind‐reading” abilities from the classic first and second order “false belief” tasks used in earlier ToM research (White et al., 2009). A key feature of narrative is the ability to provide information to the audience about the motivation, emotional responses, and psychological states of the characters (Mar, 2004). Children and adolescents who find it challenging to infer the mental states of others may struggle to reflect these aspects in their spoken narratives (Capps et al., 2000). In pragmatic terms, such narrators might also have difficulty taking the needs of their listener into account and anticipating which elements of background information are necessary for them to make sense of the story (Loveland et al., 1990).

There is considerable evidence that autistic individuals respond differently to neurotypical people in mentalising tasks, across the developmental trajectory (e.g., Baron‐Cohen et al., 1997; Kaland et al., 2008; White et al., 2009). However, the assertion that mentalising may provide a general cognitive explanation for autism attracts increasing criticism on both empirical and epistemological grounds (Gernsbacher & Yergeau, 2019), with many researchers rejecting the “deficit” model of autism (Astle & Fletcher‐Watson, 2020). Recent research into mentalising has also called into question the notion that difficulties or differences in discerning the mental states of others are uniquely associated with autism. Cotter et al. (2018) carried out a systematic review of meta‐analyses that examined social cognition, including mentalising, in different clinical populations. These authors concluded that difficulties with mentalising tasks were found across a wide range of psychiatric, neurological, and developmental conditions, reflecting a broader shift within psychological research away from categorical labels and towards a more transdiagnostic approach. It has become apparent that different diagnostic labels may have similar underlying factors (Caspi & Moffitt, 2018) and that specific diagnoses do not necessarily correspond to consistent behavioral presentations (Jones et al., 2021). Moreover, there is an increasing body of evidence to suggest that there is considerable individual variation in mentalising ability even within the neurotypical population (Devine, 2021).

Previous research has demonstrated associations between performance on mentalising tasks and specific aspects of narrative ability. For example, studies focusing on autistic participants have found scores on various mentalising measures to be significantly correlated with the use of mental state terms, syntactic diversity, and evaluation (Capps et al., 2000); and to predict narrative coherence and cohesion in story retellings (Hilvert et al., 2016). Relatively weaker mentalising ability has also been linked to increased difficulties in autistic young people's ability to accurately reproduce temporal and causal order in generalised event narratives (Loth et al., 2007). Research including non‐autistic comparison groups has also demonstrated significant relationships between mentalising and narrative skills regardless of participants' diagnostic status, in relation to mental state terms (Kuijper et al., 2017); emotional descriptors (Siller et al., 2014); and other measures of discourse pragmatics, such as referencing and causal conjunctions (Kuijper et al., 2017). At the macrostructural level, mentalising ability has been shown to be positively associated with the production of more coherent, structured, and elaborative written narratives, by both autistic and non‐autistic young people alike (Hilvert et al., 2020). However, despite providing evidence that mentalising ability is important for adolescents' narrative generation, few previous studies have also considered the role of EF within the same sample. There is also little prior work that has investigated associations between these underlying cognitive skills and the coherence of spoken accounts by autistic and non‐autistic young people.

## EXECUTIVE FUNCTION

EF is an umbrella term encompassing a range of higher‐order cognitive processes that are considered essential for “complex, novel and goal‐oriented behaviours” (Jones et al., 2018), although there is some disagreement over how best to characterise this domain of cognitive functioning. Miyake et al. (2000) proposed a triadic model that identified the core components of EF as “updating of working memory” (WM), “response inhibition” and “set shifting.” This is arguably the most replicated and empirically supported EF framework (Jewsbury et al., 2016), with its core structure successfully replicated in children (Lehto et al., 2003) and across the adult lifespan (Fisk & Sharp, 2004; Vaughan & Giovanello, 2010). Fisk and Sharp (2004) have also argued for including generativity as a fourth key function, since this was found to load onto a distinct factor in their model. Although research evidence demonstrates executive functioning differences in autistic individuals (see Demetriou et al., 2018, for a meta‐analysis), individual research findings relating to EF and autism are inconsistent. Previous studies broadly confirm difficulties with shifting tasks, while evidence for difficulties with WM, inhibition and generativity is more equivocal (Pennington & Ozonoff, 1996). However, there are notable issues with measuring EF related to unusually spiky profiles (Christ et al., 2011); task administration formats (Kenworthy et al., 2008) and the highly heterogeneous nature of the autistic population (Mottron, 2004; Sergeant et al., 2002).

Research indicates that EF may be implicated in narrative structure; for instance, in the ability to structure the narrative around key plot elements, organise these in a coherent manner, and stay on topic by ignoring irrelevant information (Ketelaars et al., 2012; Mar, 2004). EF have also been linked specifically to narrative coherence (Bourke et al., 2020; Dealy et al., 2019; Kuijper et al., 2017; Scholtens et al., 2014). Autistic individuals tend to have difficulties with complex executive tasks (Hill, 2004; Sergeant et al., 2002), which could affect the ability to organise and relate the content of a narrative. Bourke et al. (2020) suggest that challenges with mental flexibility might disadvantage autistic narrators in generating invented stories or embellishing events. However, empirical evidence for the contribution of EF to narrative ability in this population is limited. Some previous studies have indicated a relationship between WM and pragmatic narrative ability in autism, possibly due to the challenge of simultaneously processing and organising linguistic information while monitoring what has already been said (Baixauli‐Fortea et al., 2019; Schuh et al., 2016). Kuijper et al. (2017) also noted that autistic children who had poorer WM capacity produced stories that were shorter and simpler but found no association between measures of inhibition and narrative ability. Conversely, Greco et al. (2023) found no relationship between WM and narrative performance in their sample but observed that children with better inhibition skills tended to produce more fluent narratives, with fewer instances of self‐repair. Overall, the contribution of specific domains of EF to narrative skills in autistic individuals is still poorly understood (Greco et al., 2023), suggesting that further exploration of this area is warranted.

### The present study

This study investigated the relationship between the cognitive factors described above and spoken narrative skills in autistic and non‐autistic adolescents with typical‐range cognitive and linguistic abilities. The research questions were:

1. Is there an association between mentalising ability or performance on EF tasks and narrative structure (“story grammar”)?
2. Is there an association between mentalising ability or performance on EF tasks and narrative coherence?
3. After accounting for mentalising and EF ability, does diagnostic status predict narrative ability?

Based on previous literature, we predicted that mentalising ability would contribute to narrative structure and narrative coherence scores across the whole sample. We also tentatively predicted associations between some aspects of EF and narrative performance. However, due to limited previous research, we were not confident in making predictions about the specific EF components involved. We anticipated that diagnostic status would predict narrative ability, that is, that group differences in the cognitive skills described above would act as a limiting factor in the performance of the autistic group.

## METHODS

### Recruitment

Ethical approval for this study was obtained from the Department of Language and Communication Science Proportionate Review Committee at City, University of London (ETH1920‐1434) on July 14, 2020. Informed consent was obtained verbally from all participants and in written form from their parents before participating in the study. Recruitment took place through social media channels, autism research networks (Autistica and the Cambridge Autism Research Database) and secondary schools. A small incentive of an online shopping voucher was offered to participants.

### Inclusion criteria

Participants included in the autistic group were required to have a formal diagnosis of autism. Diagnostic status was supported by administering the Social Responsiveness Scale (SRS‐2; Constantino & Gruber, 2012), a parental report screening instrument which identifies the presence and level of autistic traits (T‐scores of 59 or below are considered within typical limits. However, participants with a formal autism diagnosis were not excluded if they scored slightly below this threshold, as lower scores could be due to measurement error, or might reflect the impact of interventions aimed at developing participants' communication and social skills). Participants in both groups were not excluded from the study due to additional diagnoses, such as dyslexia or ADHD. Participants were excluded if they did not reside in the United Kingdom; did not fall within the stipulated age range at the time of assessment; did not speak English fluently; or were not able to communicate verbally in full sentences. To ensure that our autistic and non‐autistic groups were comparable, participants were also excluded if they scored lower than 2 SD below the mean on the “Matrix Reasoning” subtest of the WASI‐II (Wechsler, 2011). This measure of nonverbal cognitive ability was used to estimate whether participants had intellectual abilities that were likely to fall within the borderline to typical range (i.e., corresponding to a full‐scale IQ score of more than 70), without subjecting them to excessive testing.

## PARTICIPANTS

### Study design

The study involved two groups: autistic adolescents aged 11–15 years and a non‐autistic comparison group. The wider study sample comprised 110 participants, and is described in Harvey et al. (2024). Due to missing data for some of the cognitive measures, the study reported in this article included 98 of these participants (44 autistic, 54 non‐autistic). Participants were matched at a group level on chronological age, sex, nonverbal cognitive ability, and scores on receptive vocabulary and expressive language measures. Participants were assessed at one timepoint on two narrative tasks and a battery of cognitive measures.

To investigate group differences, participants were compared on key background variables using independent samples *t*‐tests (Table 1). No significant differences were found for age, nonverbal cognitive ability, receptive vocabulary, or expressive language skills; however, *p*‐values for nonverbal cognitive ability and expressive language did not meet the threshold recommended for group matching by Mervis and Klein‐Tasman (2004). All background variables were therefore controlled in subsequent analyses. As anticipated, scores on the SRS‐2 differed significantly between the groups, reflecting their diagnostic status (*p* < 0.001). The groups did not differ significantly in terms of sex, as demonstrated by a chi‐square test (*χ2* (1) = 0.934, *p* = 0.334).

**TABLE 1:** Mean (SD) scores and ranges for background variables (age, nonverbal cognitive ability, receptive vocabulary, and expressive language) and SRS‐2 scores for autistic and non‐autistic groups, with group differences.

Variables	Autistic group (*N* = 44; 31 M, 13F)		Non‐autistic group (*N* = 54; 32 M, 22F)		Group differences
	M (SD)	Range	M (SD)	Range	
Age (months)	159.98 (16.75)	132–191	158.56 (15.96)	133–190	*t*(96) = −0.43, *p* = 0.669
Wechsler Abbreviated Scale of Intelligence—II: *Matrix Reasoning* (T‐scores: *M* = 50, SD = 10)	50.82 (9.05)	32–71	53.96 (10.05)	38–77	*t*(96) = 1.61, *p* = 0.111
British Picture Vocabulary Scale −3 (standardised scores: *M =* 100, SD = 15)	102.73 (15.47)	70–135	104.76 (12.36)	84–131	*t*(96) = 0.72, *p* = 0.471
Clinical Evaluation of Language Fundamentals −5 UK: Recalling Sentences (scaled scores: *M* = 10, SD = 3)	10.14 (2.70)	5–16	10.69 (3.43)	5–19	*t*(96) = 0.87, *p* = 0.389
Social Responsiveness Scale −2 (T‐scores: *M* = 50, SD = 10)	77.95 (10.48)	50–90	51.89 (11.74)	39–84	*t*(96) = −11.47, *p < 0.001* ***

*** < 0.001.

Data were collected from parents on participants' ethnicity and any additional neurodevelopmental or psychiatric diagnoses. Both groups comprised mostly white participants, although the autistic group was more ethnically diverse overall. There were some participants in both groups with additional diagnoses, (e.g., ADHD, Dyslexia); however, these were more common amongst the autistic participants (see supplementary materials for detailed demographics).

### Procedure

Due to the impact of the Covid pandemic and the ensuing national lockdowns, all data collection for the study was carried out online over Zoom. Although most of the assessment measures used were not designed to be administered remotely, research evidence indicates that online video‐based administration of formal language assessments shows good reliability and validity when compared to face‐to‐face administration (Ciccia et al., 2011; Waite et al., 2010). Remote administration of cognitive assessments, including EF tasks, has also been shown to produce comparable results to in‐lab administration (Collins et al., 2022). Despite this, the remote administration of assessments could potentially be challenging for some participants, particularly those with additional support needs. However, none of the autistic participants in the present study (who had low support needs) experienced difficulties with this procedure. In fact, several individuals provided positive feedback that the online assessment method corresponded to their preferences, for reasons such as being in a familiar environment and being able to switch off their camera if they wished.

Ninety‐five participants were assessed at their homes. Parents remained present during the sessions but were instructed not to prompt their child. Three of the autistic participants were assessed at school, supported by a member of staff; however, these sessions were otherwise identical in format, and all participants were assessed in a quiet room, with one adult present. Assessment sessions were led by the first author, a qualified speech and language Therapist with extensive experience of working with autistic clients. Sessions lasted between 60 and 90 min, with breaks offered to participants as often as desired. Two participants opted to complete their assessments across two shorter sessions (30–45 min). A visual timetable was used to introduce each activity. To ensure an identical procedure, the assessor followed a script, and tasks were presented in the same order for each participant.

### Background measures

Participants were assessed on their nonverbal cognitive skills using the WASI‐II: “Matrix Reasoning” subtest (Wechsler, 2011). This is a measure of “perceptual reasoning,” in which participants complete a matrix by choosing the correct picture and is scored using T‐scores (internal consistency of 0.86–0.87; test–retest stability of 0.76–0.81). Receptive vocabulary was assessed using the British Picture Vocabulary Scale (BPVS‐3; Dunn & Dunn, 2009), a standardised measure that requires participants to choose the picture that best represents a spoken word from a choice of four (reliability of 0.91). The published procedure was followed for both measures, except that stimuli were presented via screen‐share and participants were instructed to say the number of their chosen response rather than point to the item. Expressive language was assessed using the Clinical Evaluation of Language Fundamentals (CELF‐5) “Recalling Sentences” subtest (Wiig et al., 2013), in which participants were asked to repeat spoken sentences and responses were scored according to the number of errors, before being converted to scaled scores (reliability of 0.82–0.90).

### Narrative task

Participants viewed two short video clips (3–4 min each). After each one, they were immediately asked to provide a free recall account of “what happened,” with no further prompts given. To reflect the content of communication in real‐life contexts, the videos were chosen to reflect familiar social situations and approximated everyday uses of narrative skills, for example, an adolescent telling an adult about their day at school. Video A was an animated sequence with no dialogue, showing a misunderstanding between two strangers. Video B was a live‐action sequence in which a secondary aged student arrives late to his English lesson and gets in trouble. Narrative retellings were recorded and transcribed following the session, then assessed for their overall structure (macrostructure). A “story grammar” framework was used, with the events of the stimulus videos coded according to seven key story elements (setting, initiating event, plan, action/attempt, consequence, internal response, and resolution). Participants' narratives were scored against this framework, and the total number of story elements was calculated. Narrative coherence was scored using a novel framework created for this study (the “6Cs”). A rating scale (0–3) was used to assess six dimensions of coherent storytelling (context, characterisation, chronology, causality, cohesion, and congruence). These scores were then summed to create a total coherence score, out of a maximum of 18. For a detailed description of the narrative task and both scoring methods, see supplementary materials. Inter‐rater reliability measures were carried out for 36% of narrative transcripts, ranging from “good” to excellent’ for narrative structure (ICC: Video A: *M* = 0.90; Video B: *M* = 0.89) and “moderate” to “good” for narrative coherence (ICC: Video A: 0.74; Video B: 0.83).

## MENTALISING TASKS

Two assessments of mentalising ability were used to obtain a composite score. These were five short “mental state” stories (“Strange Stories”; Happé, 1994, reproduced in White et al., 2009) and a complementary, film‐based version of this task (“Silent Film Task,” Devine & Hughes, 2013). Participants were presented with the written story text accompanied by an audio recording of the researcher reading this aloud and finishing with a “mentalising” question (e.g., “Why did the burglar do that?”). Participants were also presented with five short clips from a classic silent film and asked to respond to questions, which required them to infer the thoughts, feelings or motivations of the characters (e.g., “Why did the men hide?”). Both measures used the same 0–2 coding system, in which a correct mental‐state response received 2 points, whereas a factual answer lacking any explicit reference to mental state received only 1 point. Participants were asked 11 mentalising questions in total, with a maximum possible score of 22.

## EF TASK BATTERY

A battery of assessments was devised to measure the four principal components of EF identified above:

### WM task: Working memory test battery for children: “Listening Recall” subtest (WMTB‐C)

(Pickering & Gathercole, 2001) Participants listened to sentences read out by the examiner and stated whether each sentence was true or false. Following this, participants recalled the final word from all sentences in serial order. The task gradually increased in difficulty, starting with one sentence and moving up to two, three, or more sentences in blocks of 6 trials. To progress to higher blocks, participants had to correctly recall all sentence final words in serial order on at least 4/6 trials within a block; scores reflected total number of trials correct.

### Inhibition task: “Red or Green?” (Tatool online—adapted from “Simon Task”)

(von Bastian et al., 2016) In this computer‐based task, red or green coloured circles appeared on the screen, either to the right‐ or left‐hand side. Participants responded by pressing a predetermined arrow key (left or right) for each colour, regardless of where the circle appeared on the screen. Following six practice trials, participants were presented with 80 randomised trials. Before each trial, a blank screen was displayed. The stimulus then appeared on either the left or the right of the screen for 2000 ms. To score the task, the total response time for congruent and incongruent trials was recorded and converted to seconds. The number of correct responses for each condition was then divided by the total response time, to give a rate of correct responses per second. The difference between these scores was calculated, allowing both reaction speed and accuracy to be expressed in one score (the “interference cost”).

### Shifting task: “Size? Alive?” (Tatool online—adapted from “Animacy/Size Shifting Task”)

(von Bastian et al., 2016) In this computer‐based task, participants pressed the left and right arrow keys to categorise hand‐drawn images by either animacy (animal or object) or size (larger or smaller than a football). Each trial was accompanied by a visual cue to remind participants of the relevant sorting dimension. Following six practice trials, each condition included 25 assessed trials. After animacy and size conditions were completed, participants completed 50 “mixed” trials (i.e., shifting between these conditions). Trials followed a set order, with stimuli remaining on‐screen until a button was pressed to move on to the next trial. The total response times for the two non‐switch blocks were summed. As for the inhibition task, an overall rate of correct responses was generated for condition, and then the difference was calculated to determine the “switch cost.”

### Generativity task: Category fluency test (adapted from Clinical evaluation of language fundamentals—fourth edition, CELF‐4 UK: “Word Associations” subtest)

(Semel et al., 2006) In this task, participants rapidly named as many items as they could think of within a given category. The researcher modelled the task, using the category “furniture.” Participants were timed for 1 min generating responses for the category “animals” and then again for “food.” Responses were transcribed and counted to produce a raw score. Repeated items or items that did not correspond to the category were not scored.

## DATA ANALYSIS

Mean scores on all study variables for each group are displayed in Table 2.

**TABLE 2:** Mean (SD) scores and ranges for study variables (narrative structure, narrative coherence, mentalising, and EF measures for autistic and non‐autistic groups, with group differences.

Variables	Autistic group (*N* = 44; 31 M, 13 F)		Non‐autistic group (*N* = 54; 32 M, 22 F)		Group differences
Narrative structure (/26)	M (SD)	Range	M (SD)	Range	
Video A	13.18 (4.40)	4–22	16.35 (4.30)	5–24	*t*(96) = 3.59, *p* < 0.001***
Video B	11.80 (3.79)	3–19	15.69 (3.71)	8–23	*t*(96) = 5.12, *p* < 0.001***
Narrative coherence (/18)					
Video A	11.77 (3.03)	3–16	13.04 (1.57)	9–16	*t*(61) = 2.51, *p = 0*.015*
Video B	11.55 (2.99)	4–16	12.91 (2.30)	6–17	*t*(79) = 2.48, *p = 0*.015*
Mentalising (/22)	13.91 (3.71)	6–20	15.46 (3.17)	8–21	*t*(96) = 2.24, *p = 0*.028*
EF measures					
WM (SS: *M =* 100, SD = 15)	106.05 (22.07)	65–144	111.50 (26.21)	59–144	*t*(96) = 1.10, *p = 0*.275
Inhibition (interference cost, ms)	0.20 (0.17)	−0.18–0.57	0.14 (0.19)	−0.27–0.81	*t*(96) = −1.56, *p = 0*.123
Shifting (switch cost, ms)	0.36 (0.19)	−0.06–0.88	0.42 (0.17)	0.04–0.84	*t*(96) = 1.64, *p = 0*.104
Generativity (total no. of items)	40.30 (11.17)	17–75	47.06 (11.43)	23–73	*t*(96) = 2.94, *p = 0*.004**

* < 0.05;

** < 0.01;

*** < 0.001.

Scores were analyzed using IBM SPSS Statistics, version 29. A preliminary review of the data included a principal components analysis (Jolliffe, 2002) of the four EF measures. Varimax rotation was used, and two factors were identified as those with Eigenvalues of >1.0 (Kaiser rule; Kaiser, 1960). Individual tasks were included in the factors if the correlation with overall factor score was *r* > 0.5 WM—Listening Recall task and Generativity—Category Fluency task loaded onto one factor (EF1) and Inhibition—“Red or Green?” task and Shifting—“Size/Alive?” task loaded onto another (EF2). These two components were used as the EF measures in all subsequent analyses. Pearson correlations were used to investigate the relationship between mentalising and EF scores, revealing low levels of correlation across these tasks (mentalising/EF1: *r* = 0.388, *p* < 0.001; mentalising/EF2: *r* = −0.001, *p* = 0.991).

In all analyses, narrative scores for Videos A and B were considered separately, due to differences in the content and presentation of both videos (Video A was a nonverbal animated sequence, whereas Video B featured real actors and dialogue). Since these stimuli placed different demands on participants in terms of processing and comprehension, combining the scores from the two videos might have obscured potentially differing patterns of results. There was a moderately large positive correlation for story grammar scores across Videos A and B (*r* = 0.648, *p* < 0.001), and a low positive correlation for coherence scores across both videos (*r* = 0.459, *p* < 0.001). This indicated that although the two video tasks captured somewhat similar narrative abilities, they were not directly comparable.

Hierarchical linear regressions were carried out for each narrative measure (i.e., narrative structure and narrative coherence were analysed separately for each stimulus video). The background (henceforth “control”) variables of age (months), nonverbal cognitive scores (WASI‐II: “Matrix Reasoning”), receptive vocabulary scores (BPVS‐3), and expressive language scores (CELF‐5: “Recalling Sentences”) were entered in the first step. In the second step, mentalising scores and the two EF measures (EF1, EF2) were entered, to determine whether these added any unique variance beyond the control variables alone. In the final step, Group was entered as a dummy variable, to investigate the impact of diagnostic status on narrative ability.

### Additional exploratory analyses

Despite the overall group difference observed on SRS‐2 scores, 13 non‐autistic participants scored above the cut‐off for “typical” limits on this measure. This raised the possibility that these individuals might either fulfill diagnostic criteria for autism or exhibit sub‐threshold autism‐like characteristics. However, research suggests that the presentation of some non‐autism‐related learning or behavioural difficulties may also result in elevated SRS‐2 scores (Cholemkery et al., 2014; Hus et al., 2013; Wigham et al., 2012). In either case, we were concerned that the presence of non‐autistic participants with elevated scores on this measure might alter our results. For this reason, we ran a parallel set of exploratory analyses with these participants excluded (*N* = 85), which are presented in supplementary materials.

## RESULTS

### Narrative structure (story grammar)

#### *Video* A *(animated, no dialogue)*

A hierarchical linear regression model was used to investigate the contribution of the control variables (age, nonverbal cognitive scores, receptive vocabulary scores and expressive language scores); mentalising and EF scores; and Group to narrative structure scores for Video A. Step 1 of the regression model (control variables) was non‐significant, *R* ^2^ = 0.085, *F*(4, 93) = 2.171, *p = 0*.078, adj. *R* ^2^ = 0.046. Step 2 of the model was statistically significant, *R* ^2^ = 0.257, *F*(7, 90) = 4.458, *p* < 0.001, adj. *R* ^2^ = 0.200, explaining an additional 17% of unique variance once the control variables had been accounted for (∆*R* ^2^ = 0.172). Mentalising was the only significant predictor of story grammar scores for Video A (*β* = 0.386, *p <* 0.001), with EF scores failing to reach significance (EF1: *β* = 0.226, *p =* 0.056; EF2: *β* = −0.083, *p =* 0.377). In the final model (Step 3), the inclusion of Group significantly predicted 4% of additional variance overall, with the autistic group showing lower scores than the non‐autistic group, ∆*R* ^2^ = 0.043, *F*(8, 89) = 4.779, *p* < 0.001 (Group: *β* = −0.222, *p =* 0.022). Mentalising remained significant as a predictor variable (*β* = 0.348, *p* = 0.001). See Table 3.

**TABLE 3:** Hierarchical linear regression predicting total narrative structure (“story grammar”) scores for Video A from control variables, mentalising and EF scores, and diagnostic group (final model).

Variables	B (95% CI)	SE B	*β*	*p*
Constant	9.175 (−2.552–20.903)	5.902	‐	0.124
Age	0.018 (−0.034–0.070)	0.026	0.064	0.491
Receptive vocabulary	−0.013 (−0.096–0.071)	0.042	−0.038	0.764
Expressive language	0.032 (−0.311–0.375)	0.173	0.022	0.853
Nonverbal ability	0.001 (−0.105–0.107)	0.053	0.002	0.986
Mentalising**	0.459 (0.186–0.732)	0.138	0.348	0.001**
EF1	0.727 (−0.350–1.804)	0.542	0.158	0.183
EF2	−0.338 (−1.171–0.496)	0.419	−0.073	0.423
Group*	−2.043 (−3.778—−0.308)	0.873	−0.222	0.022*

*Note*: *N* = 98. *R* ^2^ = 0.085 for Step 1 (*p* = 0.078). ∆*R* ^2^ = 0.172 for Step 2 (*p* < 0.001***). ∆*R* ^2^ = 0.043 for Step 3 (*p <* 0.001***).

* < 0.05;

** < 0.01.

### Narrative structure (“story grammar”)

#### *Video* B *(real actors, with dialogue)*

A hierarchical linear regression model was used to investigate the contribution of the control variables (age, nonverbal cognitive scores, receptive vocabulary scores, and expressive language scores); mentalising and EF scores; and Group to narrative structure scores for Video B. Step 1 of the regression model was statistically significant, *R* ^2^ = 0.113, *F*(4, 93) = 2.970, *p = 0*.023, adj. *R* ^2^ = 0.075, although none of the control variables were individually significant. Step 2 of the model was also significant, *R* ^2^ = 0.261, *F*(7, 90) = 4.547, *p* < 0.001, adj. *R* ^2^ = 0.204, explaining an additional 15% of unique variance once the control variables had been accounted for (∆*R* ^2^ = 0.148). Mentalising was the only significant predictor of “story grammar” scores for Video B (*β* = 0.421, *p <* 0.001), with EF scores failing to reach significance (EF1: *β* = 0.028, *p =* 0.811; EF2: *β* = −0.111, *p =* 0.237). In the final model (Step 3), the inclusion of Group significantly predicted 13% of additional variance overall, with the autistic group showing lower scores than the non‐autistic group, ∆*R* ^2^ = 0.133, *F*(8, 89) = 7.240, *p* < 0.001 (Group: *β* = −0.390, *p <* 0.001). As for Video A, mentalising remained significant as a predictor variable (*β* = 0.354, *p <* 0.001). See Table 4.

**TABLE 4:** Hierarchical linear regression predicting total narrative structure (“story grammar”) scores for Video B from control variables, mentalising and EF scores, and diagnostic group (final model).

Variables	B (95% CI)	SE B	*β*	*p*
Constant	3.651 (−6.310–13.612)	5.013	‐	0.468
Age	0.031 (−0.013–0.075)	0.022	0.120	0.166
Receptive vocabulary	0.016 (−0.055–0.087)	0.036	0.051	0.663
Expressive language	0.106 (−0.185–0.397)	0.147	0.079	0.471
Nonverbal ability	0.021 (−0.069–0.111)	0.045	0.048	0.646
Mentalising***	0.426 (0.194–0.658)	0.117	0.354	<0.001***
EF1	−0.387 (−1.301–0.528)	0.460	−0.092	0.403
EF2	−0.396 (−1.104–0.312)	0.356	−0.094	0.270
Group***	−3.278 (−4.752–1.804)	0.742	−0.390	<0.001***

*Note*: *N* = 98. *R* ^2^ = 0.113 for Step 1 (*p* = 0.023*). ∆*R* ^2^ = 0.148 for Step 2 (*p* < 0.001***). ∆*R* ^2^ = 0.133 for Step 3 (*p <* 0.001***).

* < 0.05;

** < 0.01;

*** < 0.001.

### Narrative coherence

#### *Video* A *(animated, no dialogue)*

A hierarchical linear regression model was used to investigate the contribution of the control variables (age, nonverbal cognitive scores, receptive vocabulary scores, and expressive language scores); mentalising and EF scores; and Group to narrative coherence scores for Video A. Step 1 of the regression model (control variables) was non‐significant, *R* ^2^ = 0.071, *F*(4, 93) = 1.772, *p =* 0.141, adj. *R* ^2^ = 0.031. Step 2 of the model was statistically significant, *R* ^2^ = 0.230, *F*(7, 90) = 3.841, *p =* 0.001, adj. *R* ^2^ = 0.170, explaining an additional 16% of unique variance once the control variables had been accounted for (∆*R* ^2^ = 0.159). Mentalising significantly predicted coherence scores for Video A (*β* = 0.335, *p =* 0.002), as did EF1 scores (*β* = 0.273, *p =* 0.024). EF2 scores were not significant at Step 2 (*β* = −0.078, *p =* 0.412). In the final model (Step 3), although the inclusion of Group significantly predicted 2% of additional variance, ∆*R* ^2^ = 0.021, *F*(8, 89) = 3.720, *p <* 0.001, Group was not individually significant (Group: *β* = −0.153, *p* = 0.122). Mentalising remained significant as a predictor at Step 3 (*β* = 0.309, *p* = 0.005), whereas EF1 scores fell short of significance (*β* = 0.226, *p* = 0.067). See Table 5.

**TABLE 5:** Hierarchical linear regression predicting total narrative coherence scores for Video A from control variables, mentalising and EF scores, and diagnostic group (final model).

Variables	B (95% CI)	SE B	*β*	*p*
Constant***	11.154 (4.803–17.505)	3.196	‐	<0.001***
Age	0.003 (−0.025–0.031)	0.014	0.018	0.851
Receptive vocabulary	0.013 (−0.032–0.058)	0.023	0.075	0.570
Expressive language	−0.003 (−0.189–0.183)	0.093	−0.004	0.976
Nonverbal ability	−0.048 (−0.105–0.010)	0.029	−0.192	0.103
Mentalising**	0.213 (0.065–0.361)	0.074	0.309	0.005**
EF1	0.545 (−0.039–1.128)	0.294	0.226	0.067
EF2	−0.173 (−0.624–0.278)	0.227	−0.072	0.448
Group	−0.739 (−1.678–0.201)	0.473	−0.153	0.122

*Note*: *N* = 98. *R* ^2^ = 0.071 for Step 1 (*p* = 0.141). ∆*R* ^2^ = 0.159 for Step 2 (*p =* 0.001**). ∆*R* ^2^ = 0.021 for Step 3 (*p* < 0.001***).

** < 0.01;

*** < 0.001.

### Narrative coherence

#### *Video* B *(real actors, with dialogue)*

A hierarchical linear regression model was used to investigate the contribution of the control variables (age, nonverbal cognitive scores, receptive vocabulary scores, and expressive language scores); mentalising and EF scores; and Group to narrative coherence scores for Video B. Step 1 of the regression model (control variables) was statistically significant, *R* ^2^ = 0.219, *F*(4, 93) = 6.519, *p < 0*.001, adj. *R* ^2^ = 0.185; with age and receptive vocabulary significantly predicting coherence scores (Age: *β* = 0.219, *p* = 0.020; receptive vocabulary: *β* = 0.265, *p* = 0.036). Step 2 of the model was also significant, *R* ^2^ = 0.316, *F*(7, 90) = 5.933, *p* < 0.001, adj. *R* ^2^ = 0.263, explaining an additional 10% of unique variance once the control variables had been accounted for (∆*R* ^2^ = 0.097). Mentalising was the only significant predictor of coherence scores for Video B at Step 2 (*β* = 0.348, *p <* 0.001), with EF scores failing to reach significance (EF1: *β* = 0.005, *p =* 0.967; EF2: *β* = −0.073, *p =* 0.418). At Step 2, age and receptive vocabulary were non‐significant (age: *β* = 0.167, *p* = 0.067; receptive vocabulary: *β* = 0.182, *p* = 0.144). In the final model (Step 3), although the inclusion of Group significantly predicted 2% of additional variance (∆*R* ^2^ = 0.024, *F*(8, 89) = 5.737, *p* < 0.001), Group was not individually significant (Group: *β* = −0.167, *p* = 0.073). However, mentalising remained significant as a predictor variable (*β* = 0.319, *p* = 0.002), and age was also significant in this final model (*β* = 0.182, *p* = 0.044). See Table 6.

**TABLE 6:** Hierarchical linear regression predicting total narrative coherence scores for Video B from control variables, mentalising and EF scores, and diagnostic group (final model).

Variables	B (95% CI)	SE B	*β*	*p*
Constant	−0.520 (−7.218–6.177)	3.371	‐	0.878
Age*	0.030 (0.001–0.060)	0.015	0.182	0.044*
Receptive vocabulary	0.041 (−0.007–0.089)	0.024	0.208	0.094
Expressive language	0.080 (−0.115–0.276)	0.099	0.093	0.417
Nonverbal ability	0.011 (−0.050–0.071)	0.030	0.039	0.725
Mentalising**	0.247 (0.091–0.404)	0.079	0.319	0.002**
EF1	−0.127 (−0.742–0.489)	0.310	−0.047	0.684
EF2	−0.178 (−0.654–0.298)	0.240	−0.066	0.459
Group	−0.906 (−1.897–0.085)	0.499	−0.167	0.073

*Note*: *N* = 98. *R* ^2^ = 0.219 for Step 1 (*p* < 0.001***). ∆*R* ^2^ = 0.097 for Step 2 (*p <* 0.001**). ∆*R* ^2^ = 0.024 for Step 3 (*p* < 0.001***).

* < 0.05;

** < 0.01;

*** < 0.001.

## DISCUSSION

The present study investigated associations between narrative ability and performance on measures of mentalising and EF in a sample of autistic and non‐autistic adolescents with comparable linguistic and nonverbal skills. In line with predictions, Mentalising was the most important variable in predicting narrative ability, whether assessed using measures of narrative structure (story grammar) or narrative coherence, across both videos. In fact, Mentalising emerged as a more consistent predictor of narrative scores than diagnostic group, remaining significant in all models even after Group was added in the final step. By contrast, contrary to predictions, EF scores did not significantly predict narrative scores in any model, once all variables were included, and this finding was consistent for measures of both narrative structure and coherence across both videos.

It was noteworthy that Group was a significant predictor of participants' narrative structure scores for both videos, with the autistic group scoring lower than the non‐autistic group, but did not predict narrative coherence scores for either video. Similarly, although age and receptive vocabulary scores predicted narrative coherence scores for Video B in some steps of the regression model, the overall pattern of results indicated that the background variables (age, nonverbal cognitive ability, receptive vocabulary, expressive language) were not key factors contributing to narrative performance in this sample of adolescents with typical‐range cognitive and verbal abilities. Additional exploratory analyses excluding non‐autistic participants with elevated SRS‐2 scores (Appendix B) confirmed our overall findings that Mentalising predicts both narrative structure and coherence, irrespective of Group.

These findings highlight the importance of mentalising skills for producing well‐structured and coherent accounts of events that take place within social contexts. Mentalising may play a role in two different ways (see Ketelaars et al., 2012): (a) helping individuals to comprehend the original events that have transpired (or, in the present study, interpreting the content of the videos); and (b) generating a verbal account of these events in order to relay the most salient details to their listener, whilst providing sufficient contextual information.

Our study strongly suggests that young people with poorer mentalising skills may struggle with providing spoken narrative accounts, regardless of their diagnostic status. The findings align with previous research in this area demonstrating associations between mentalising ability and various aspects of narrative performance in autistic samples (e.g., Capps et al., 2000; Hilvert et al., 2016; Loth et al., 2007) and in samples including both autistic and non‐autistic children (Hilvert et al., 2020; Kuijper et al., 2017; Siller et al., 2014). Our findings also uphold recent work indicating that individual differences in mentalising abilities transcend diagnostic categories (e.g., Cotter et al., 2018; Devine, 2021).

Neither of the dimensions of EF measured in this study were found to significantly predict either narrative structure or coherence in our sample. It is possible, however, that the large number of predictor variables may have meant that we were unable to detect small, but real, effects for some of these aspects of EF in relation to narrative scores. An alternative explanation could be that the experimental tasks used in the present study might not accurately reflect how participants use their executive skills in “real‐life” contexts (see Kenworthy et al., 2008).

Another speculative explanation for these null findings is that our sample may have been comprised of young people with relatively strong EF skills, even within the autistic group. The practical requirements of participation in the study, such as the ability to focus during a 1 h assessment session, could have introduced a sampling bias whereby adolescents with poorer EF skills were less likely to volunteer. Our recruitment strategy, which principally targeted adolescents attending mainstream educational settings, could have also inadvertently “screened out” participants with more pronounced executive difficulties who might struggle to cope with the demands of a mainstream secondary school (see Jacobson et al., 2011) and therefore might be more likely to attend specialist or alternative provisions. Since we cannot discount the possibility that our sample was not representative of EF skills within the wider autistic population, we consider that further research in this area is warranted to explore the potential impact of executive difficulties on spoken discourse.

While Group was a significant predictor for narrative structure scores across both videos, with autistic adolescents scoring lower than their non‐autistic counterparts, mentalising remained significant even after Group was added to the regression model in the final step. In relation to narrative coherence, however, Group did not significantly predict additional variance in scores for either video when entered in the final step of the regressions (after controlling for all other variables). This suggests that telling coherent stories might be an area of relative strength for autistic narrators. These findings reflect a broader shift in developmental research towards a neurodiversity perspective, with increasing recognition that there can be common areas of strength and challenge between individuals who may or may not have a particular diagnostic label. Astle and Fletcher‐Watson (2020) argue that rather than focusing on diagnoses, the aim of research should be “to establish which dimensions are important for understanding individual outcomes, irrespective of the diagnostic category applied” (p. 432). Our study findings indicate that adolescents' narrative abilities should not be assumed based on diagnosis (or lack thereof) and suggest that supporting and facilitating mentalising skills might be a useful approach for any young person who struggles with spoken discourse. The findings also lend support to previous research linking mentalising ability in older children and adolescents to broader social outcomes (Bosacki & Wilde Astington, 1999; Devine & Hughes, 2013), since challenges with narrative skills are known to have a negative impact upon young people's social competence (see Petersen et al., 2014).

## LIMITATIONS

The autistic adolescents included in our sample demonstrated broadly typical‐range cognitive and linguistic skills and presented with low support needs. This means that the findings may not be generalisable to the wider autistic population, who may have a co‐occurring intellectual disability, language disorder, or other complex support needs. Although groups were matched on participant sex, the overall sample was not balanced in terms of male and female participants. This resulted from difficulties recruiting enough autistic girls to the study, a common issue in autism research (Shefcyk, 2015). Since research indicates that autistic girls may present with a different linguistic profile to boys (e.g., Conlon et al., 2019), the comparative lack of female participants may limit the generalisability of our findings.

To provide a representative sample of the broader population, no participants in either group were excluded because of additional diagnoses, such as Dyslexia. However, many more participants in the autistic group had co‐occurring diagnoses than participants in the non‐autistic comparison group, particularly ADHD (see Appendix A). This reflects the high prevalence of multiple diagnoses amongst autistic people, with autism/ADHD being a common dual diagnosis (Stevens et al., 2016). This may introduce a potentially confounding variable when interpreting the results of this study, and the impact of co‐occurring conditions on narrative ability warrants further study.

A further limitation of the present study was that only a “moderate” to “good” level of inter‐rater reliability was achieved for the “6Cs” coherence framework. We note that this novel assessment tool requires further piloting and refinement to reduce subjectivity in scoring and improve its reliability.

In conclusion, mentalising skills appear to be fundamental in supporting adolescents' production of well‐structured and coherent verbal narrative accounts, regardless of whether they have an autism diagnosis or not. Further research in this area could consider the factors contributing to poorer mentalising ability, and whether developing adolescents' mentalising skills might improve their ability to successfully generate spoken narratives in their daily lives.

## ETHICS STATEMENT

Ethical approval for this study was obtained from the Department of Language and Communication Science Proportionate Review Committee at City, University of London (ETH1920‐1434).

## Supporting information


**Data S1.** Supplementary Information.

## Data Availability

Data is not publicly available due to ethical restrictions.
