# Supplementary material for: Narrative abilities of autistic and non‐autistic adolescents: The role of mentalising and executive function
Source: Autism Res. 2024 Nov 19;18(1):152–65. doi: 10.1002/aur.3272 (PMC11782712; doi:10.1002/aur.3272)
Supplement: Supplementary file 1 — Data S1. Supplementary Information. [file AUR-18-152-s001.docx]

**Appendices**

**A:**  Additional demographic information

**B:**  Narrative structure (story grammar) scoring frameworks

**C:**  Narrative coherence (‘6Cs’) scoring framework

**D:**  Correlations between study variables

**E:**  Exploratory analyses excluding non-autistic participants with elevated SRS-2 scores

**Appendix A: Additional demographic information**

**Ethnicity of study participants (by parent report):**

| **Autistic group (N= 44)** | **Non-autistic group (N= 54)** |
| --- | --- |
| White British: 32 (72.7%)  White Other: 7 (15.9%)  White and Black Caribbean: 1 (2.3%)  Black British: 1 (2.3%)  Black Caribbean: 1 (2.3%)  Asian - Bangladeshi: 1 (2.3%)  Other – Kurdish: 1 (2.3%) | White British: 46 (85.2%)  White Other: 4 (7.4%)  White and Black Caribbean 1 (1.9%)  Black Caribbean: 1 (1.9%)  Asian (Pakistani): 1 (1.9%)  Other – Arab: 1 (1.9%) |

**Number of study participants with neurodevelopmental or psychiatric diagnoses other than autism (by parent report):**

| **Autistic group (N=44)** | **Non-autistic group (N=54)** |
| --- | --- |
| ADHD:12 (27.3%)  Dyslexia: 3 (6.8%)  Dyspraxia/DCD: 3 (6.8%)  Sensory Processing Disorder: 2 (4.5%)  Anxiety: 1 (5.7%)  Mild Learning Difficulties: 1 (2.3%)  Dysgraphia: 1 (2.3%)  Epilepsy: 1 (2.3%) | Dyslexia: 5 (9.3%)  ADHD: 2 (3.7%)  Dyspraxia/DCD: 2 (3.7%)  Anxiety: 1 (1.9%) |

**Appendix B: Narrative structure (story grammar) scoring framework**

| **Story grammar element:** | **Score:** |
| --- | --- |
| Setting (S) |  |
| Initiating event (IE) |  |
| Plan (P) |  |
| Action/Attempt (A) |  |
| Consequence (C) |  |
| Internal response (IR) |  |
| Resolution (R) |  |
| **Story grammar total score:** |  |

**Video A:**

| **Main story events** | **Story grammar code** | **Included in narrative? (0/1)** |
| --- | --- | --- |
| **Episode 1** |  |  |
| An old lady/woman/grandma | **S** |  |
| At the train station | **S** |  |
| She wants to get some biscuits from the vending machine/wants to buy a snack/wants something to eat | **P** |  |
| She puts some money in the machine | **A** |  |
| The packet of biscuits gets stuck/won’t come out | **IE** |  |
| She is annoyed/frustrated/angry | **IR** |  |
| She hits/kicks/bangs the vending machine | **A** |  |
| The packet of biscuits comes out/falls down | **C** |  |
| She is pleased/happy | **IR** |  |
| She takes the biscuits/puts the packet in her bag | **R** |  |
| **Episode 2** |  |  |
| The old lady goes into the train station/onto the platform/sits down on a bench/ sits next to a teenager/man/boy/ | **S** |  |
| She opens the packet to eat a biscuit/she starts eating the biscuits | **P** |  |
| The teenager takes a biscuit/also starts eating the biscuits | **IE** |  |
| She is annoyed/frustrated/angry/  She thinks he is eating her biscuits | **IR** |  |
| He eats another biscuit/keeps taking the biscuits | **A** |  |
| The old lady tells the teenager off/shouts at him | **C** |  |
| They fight over the biscuits/she tries to take the last biscuit off him | **A** |  |
| The teenager offers half of the last biscuit to the old lady/tries to share the last biscuit with her | **C** |  |
| The old lady crumbles the biscuit in her hand/throws it down/rejects his offer | **R** |  |
| **Episode 3** |  |  |
| The old lady gets on the train | **S** |  |
| The ticket inspector comes/she needs to show her ticket/she gets out her ticket | **IE** |  |
| She opens her bag/she puts the ticket away/she looks in her bag | **A** |  |
| She sees her (unopened) packet of biscuits in the bag | **C** |  |
| The old lady realises she was eating the teenager’s biscuits/realises that he was actually being generous | **IR** |  |
| The old lady is regretful/sorry/feels bad for getting angry with him | **IR** |  |
| The train leaves/The teenager walks away/puts the empty packet in the bin | **R** |  |

**Video B:**

| **Main story events** | **Story grammar code** | **Included in narrative? (0/1)** |
| --- | --- | --- |
| **Episode 1** |  |  |
| A boy/Joe/ a group of boys/Year 7s | **S** |  |
| Playing football/outside/on the playing field/at breaktime. | **S** |  |
| The bell rings/it’s time to go to class/Joe’s friends say they need to go | **IE** |  |
| Joe keeps playing/falls over/gets tackled | **A** |  |
| He gets mud on his shirt/gets his uniform dirty | **C** |  |
| Joe’s friend offers his water bottle to clean the shirt/his friend tries to help | **A** |  |
| Joe refuses/says no/doesn’t want it to look like he’s wet himself | **A** |  |
| Joe is worried about his teacher’s reaction/doesn’t want to get in trouble | **IR** |  |
| He decides to clean his shirt before going to his lesson | **P** |  |
| **Episode 2** |  |  |
| Joe goes into the school/is in the school corridor/hallway | **S** |  |
| He is lost/ he can’t find his classroom/ he doesn’t know where to go | **IE** |  |
| He asks a group of girls for directions to his classroom | **P** |  |
| The girls give him the wrong directions/make fun of him | **A** |  |
| Joe is fed up/upset/disappointed | **IR** |  |
| An older student approaches Joe and tells him where to go / Someone else helps him by telling him the right directions. | **R** |  |
| **Episode 3** |  |  |
| Joe goes to his classroom/enters the classroom | **S** |  |
| He is late to the lesson /The teacher/Mr Drew asks Joe why he is late | **IE** |  |
| Joe tries to apologise/says sorry | **P** |  |
| Mr Drew makes fun of Joe in front of the class/ Mr Drew is mean to Joe/ The teacher tells him off | **A** |  |
| The whole class laughs at Joe | **C** |  |
| Joe is annoyed/angry/upset | **IR** |  |
| Joe mutters “get lost” to the teacher/tells Mr Drew to “get lost” | **A** |  |
| Mr Drew is cross/annoyed | **IR** |  |
| Mr Drew asks Joe to repeat what he said | **C** |  |
| Joe pretends he said that he “got lost” | **A** |  |
| Mr Drew tells Joe to sit down/ Joe goes to his seat. | **R** |  |

**Appendix C: Narrative coherence (‘6Cs’) scoring framework**

|  | **0** | **1** | **2** | **3** |
| --- | --- | --- | --- | --- |
| **Context** | No attempt to provide any information about the time setting or location of the events described. | Some aspect of setting is mentioned during the narrative, but lacks specificity (e.g., "they're outside"; "it’s late")  **OR:** Narrator provides enough contextual information that the setting can be correctly inferred (minimum of TWO contextual clues). | Narrator provides specific information about either location or time setting (e.g. "at the train station"; “in the classroom”, "it's break-time")  **OR**: Narrator provides non-specific information about both location and time. | Narrator orients the listener to both the location and the time setting (and at least one of these is specific). |
| **Characterisation** | Characters are underdeveloped. No use of internal state terms in the narrative (i.e., cognitive or emotional) | Some limited information is provided about the motivations of the characters (i.e., at least one internal state term is used in the narrative to describe thoughts or feelings). | A range of internal state terms are used in the narrative to describe thoughts or feelings. The motivations of the main or supporting characters are evident. | More than one character in the narrative is well-developed, with a range of internal state terms providing information about their thoughts or feelings. The motivations of the main and/or supporting characters are evident. |
| **Chronology** | Story events do not follow a logical chronological sequence. | The narrative follows a logical chronological sequence, but a significant number of story events are omitted (less than 10 main events reported in total). | Story events follow a logical chronological sequence, with any timeline violations explained by the narrator. However, the narrative ends abruptly and lacks a clear resolution. | Story events follow a logical chronological sequence, with any timeline violations explained by the narrator. The narrative includes a clear ending or resolution. |
| **Causality** | No attempt to link cause and effect in the narrative. Even if story events are listed in the correct order, it is not clear how they are related (e.g., consequences are presented without the causes being mentioned). | There are some identifiable instances of cause and effect in the narrative (e.g., characters’ actions are followed by direct consequences). However, the narrator does not use any causal conjunctions to highlight these relationships. | The narrator attempts to highlight cause and effect relationships between story events. At least one causal conjunction is used in the narrative (e.g., 'so', 'because', 'since', 'as', 'even though', 'consequently', 'therefore', 'as a result'...) | The narrator uses a range of causal conjunctions to explain cause and effect relationships between key story events (e.g., 'so', 'because', 'since', 'as', 'even though', 'consequently', 'therefore', 'as a result'...) |
| **Cohesion** | Referencing is unclear, making the story difficult to follow. | Referencing is somewhat unclear, but despite the presence of ambiguous references, the listener is still able to follow the story easily. | Referencing is generally clear, with no more than two ambiguous references. | Referencing is accurate throughout the entire story; (i.e., all pronouns can be traced back to an appropriate antecedent). |
| **Congruence** | Off-topic, bizarre or extraneous utterances impede comprehension of the story. | Presence of more than one off-topic, bizarre or extraneous utterance; however, these comments do not impede overall comprehension of the story. | Utterances are generally on-topic, with not more than one off-topic or bizarre remark. | All utterances are pertinent to the events being described in the narrative. |

**Appendix D: Correlations between study variables**

|  | **1.** | **2.** | **3.** | **4.** | **5.** | **6.** | **7.** | **8.** |
| --- | --- | --- | --- | --- | --- | --- | --- | --- |
| **1. Structure (video A)** | ***-*** |  |  |  |  |  |  |  |
| **2. Structure (video B)** | ***r*= .648, *p*<.001***** | - |  |  |  |  |  |  |
| **3. Coherence (video A)** | ***r*= .613, *p*<.001***** | ***r*= .475, *p*<.001***** | - |  |  |  |  |  |
| **4. Coherence (video B)** | ***r*= .414, *p*<.001***** | ***r*= .689, *p*<.001***** | ***r*= .459, *p*<.001***** | - |  |  |  |  |
| **5. Mentalising** | ***r*= .456,** ***p*<.001***** | ***r*= .480, *p*<.001***** | ***r*= .395, *p*<.001***** | ***r*= .488, *p<*.001***** | - |  |  |  |
| **6. WM** | ***r*= .273,** ***p=* .007**** | *r*= .196, *p=*.053 | ***r*= .346, *p*<.001**** | ***r*= .282, *p=*.005**** | ***r*= .333, *p<*.001***** | - |  |  |
| **7. Inhibition** | *r*= -.132, *p=* .195 | *r*= -.174, *p=*.087 | *r*= -.097, *p=*.344 | *r*= -.195, *p=*.054 | *r*= -.119, *p=*.242 | *r*= -.056, *p=*.586 | - |  |
| **8. Shifting** | *r*= .068, *p=*.505 | *r*= .050, *p=*.622 | *r*= .077, *p=*.453 | *r*= .138, *p=*.176 | *r*= .191, *p=*.060 | *r*= .194, *p=*.055 | *r*= .093, *p=*.360 | - |
| **9. Generativity** | ***r*= .298, *p=*.003**** | ***r*= .213, *p=*.035*** | ***r*= .261, *p=*.009**** | *r*= .158, *p=*.119 | ***r*= .258, *p=*.010*** | ***r*= .375, *p<*.001***** | *r*= -.130, *p=*.201 | *r*= .136, *p=*.183 |

**Appendix E:**

**Exploratory analyses excluding non-autistic participants with elevated SRS-2 scores (N=13)**

**Table 1. Mean (SD) scores for control variables (age, non-verbal cognitive ability, receptive vocabulary and expressive language) and SRS-2 scores for autistic and non-autistic groups, with group differences.**

| **Variables** | **Autistic group (N=44)** | **Non-autistic group (N=41)** | **Group differences** |
| --- | --- | --- | --- |
| **Age**  (months) | 159.98 (16.75) | 158.32 (16.21) | *t*(83)= -.46, *p*= .644 |
| **Non-verbal ability:**  WASI-II - Matrix Reasoning (T-scores: *M*= 50, SD= 10) | 50.82 (9.05) | 54.78 (9.68) | *t*(83)= 1.95, *p*= .054 |
| **Receptive vocabulary:**  BPVS-3 (standardised scores: *M=* 100, SD= 15) | 102.73 (15.47) | 106.78 (11.86) | *t*(83)= 1.35, *p*= .181 |
| **Expressive language:**  CELF-5 UK - Recalling  Sentences (scaled scores: *M*= 10, SD= 3) | 10.14 (2.70) | 11.61 (3.27) | *t*(83)= 2.27, *p*= .026* |
| **Autistic traits:**  Social Responsiveness Scale-2 (T-scores: *M*= 50, SD= 10)  *Note: T-scores >59 are indicative of social communication difficulties* | 77.95 (10.48) | 46.15 (5.13) | *t*(83)= -17.95, *p<* .001*** |

**Narrative structure (story grammar)**

**Video A**

Step 1 of the regression model (control variables) was statistically significant, R^2^= .115, *F*(4, 80)= 2.607, *p=* .042, adj. R^2^= .071, although expressive language was the only individual variable that was significant (β= .253, *p=* .046)*.*

Step 2 of the model was also statistically significant, R^2^= .319, *F*(7, 77)= , *p*< .001, adj. R^2^= .257, explaining an additional 20% of unique variance once the control variables had been accounted for (∆R^2^=.203). Mentalising significantly predicted story grammar scores for Video A (β= .434, *p*< .001), as did EF1 scores (β= .283, *p=* .024). However, EF2 scores were not significant (β= -.025, *p=* .793).

In the final model (Step 3), the inclusion of Group significantly predicted 2% of additional variance overall, ∆R^2^=.019, *F*(8, 76)= , *p*< .001, although Group was not an individually significant predictor (Group: β= -.151, *p=* .146). Mentalising remained significant as a predictor variable (β= .410, *p*< .001), but EF1 did not retain its significance in this final step (β= .230, *p=* .074). See **Table 2**.

**Table 2. Hierarchical multiple regression predicting total narrative structure (‘story grammar’) scores for Video A from control variables, Mentalising and EF scores, and diagnostic group (final model).**

| **Variables** | **B (95% CI)** | **SE B** | **β** | ***p*** |
| --- | --- | --- | --- | --- |
| Constant  Age  Receptive vocabulary  Expressive language  Non-verbal ability  Mentalising**  EF1  EF2  Group* | 9.772 (-3.104 - 22.648)  .022 (-.033 - .078)  -.039 (-.126 - .048)  .064 (-.309 - .438)  -.021 (-.133 - .090)  .530 (.242 - .818)  1.046 (-.103 - 2.195)  -.112 (-.966 - .742)  -1.392 (-3.279 - .496) | 6.465  .028  .044  .187  .056  .145  .577  .429  .948 | -  .079  -.117  .043  -.044  .410  .230  -.025  -.151 | .135  .424  .376  .733  .703  <.001***  .074  .794  .146 |

Note: N=85. R^2^= .115 for Step 1 (*p*=.042*). ∆R^2^= .203 for Step 2 (*p*< .001***). ∆R^2^ = .019 for Step 3 (*p<* .001***).

**Video B**

Step 1 of the regression model (control variables) was statistically significant overall, R^2^= .119, *F*(4, 80)= 2.702, *p=* .036, adj. R^2^= .075, although none of the control variables were individually significant.

Step 2 of the model was also significant, R^2^= .286, *F*(7,77)= 4.397, *p*< .001, adj. R^2^= .221, explaining an additional 17% of unique variance once the control variables had been accounted for (∆R^2^=.167). Mentalising was the only significant predictor of story grammar scores for Video B (β= .449, *p*< .001), while EF scores were not significant (EF1: β= .125, *p=* .322; EF2: β= -.062, *p=* .533).

In the final model (Step 3), the inclusion of Group significantly predicted 12% of additional variance overall, ∆R^2^=.119, *F*(8, 76)= 6.459, *p*< .001 (Group: β= -.381, *p*< .001), with Mentalising remaining significant as a predictor variable (β= .389, *p*< .001). See **Table 3**.

**Table 3. Hierarchical multiple regression predicting total narrative structure (‘story grammar’) scores for Video B from control variables, Mentalising and EF scores, and diagnostic group (final model).**

| **Variables** | **B (95% CI)** | **SE B** | **β** | ***p*** |
| --- | --- | --- | --- | --- |
| Constant  Age  Receptive vocabulary  Expressive language  Non-verbal ability  Mentalising***  EF1  EF2  Group*** | 7.914 (-3.147 - 18.975)  .019 (-.028 - .067)  .002 (-.073 - .077)  .045 (-.276 - .366)  .003 (-.093 - .099)  .456 (.208 - .704)  -.027 (-1.014 - .960)  -.247 (-.981 - .486)  -3.176 (-4.797 - -1.554) | 5.554  .024  .037  .161  .048  .124  .496  .368  .814 | -  .076  .007  .033  .007  .389  -.007  -.061  -.381 | .158  .421  .958  .780  .949  <.001***  .957  .504  <.001*** |

Note: N=85. R^2^= .119 for Step 1 (*p*=.036*). ∆R^2^= .167 for Step 2 (*p*< .001***). ∆R^2^ = .119 for Step 3 (*p<* .001***).

**Narrative coherence**

**Video A**

Step 1 of the regression model (control variables) was non-significant, R^2^= .078, *F*(4, 80)= 1.686, *p*= .161, adj. R^2^= .032.

Step 2 of the model was statistically significant, R^2^= .275, *F*(7, 77)= 4.179, *p*< .001, adj. R^2^= .209, explaining an additional 20% of unique variance once the control variables had been accounted for (∆R^2^=.198). Mentalising significantly predicted narrative coherence scores for Video A (β= .385, *p*= .001), as did EF1 scores (β= .332, *p=* .010). However, EF2 scores were not significant (β= -.073, *p=* .465).

In the final model (Step 3), the inclusion of Group significantly predicted 2% of additional variance overall, ∆R^2^=.019, *F*(8, 76)= 3.954, *p*< .001, although Group was not an individually significant predictor (Group: β= -.150, *p=* .161). Mentalising and EF1 scores both remained significant as predictor variables (Mentalising: β= .361, *p*= .003; EF1: β= .280, *p*= .036). Non-verbal cognitive ability also significantly predicted narrative coherence scores in this final model (β= -.249, *p*= .040). See **Table 4**.

**Table 4. Hierarchical multiple regression predicting total narrative coherence scores for Video A from control variables, Mentalising and EF scores, and diagnostic group (final model).**

| **Variables** | **B (95% CI)** | **SE B** | **β** | ***p*** |
| --- | --- | --- | --- | --- |
| Constant***  Age  Receptive vocabulary  Expressive language  Non-verbal ability*  Mentalising**  EF1*  EF2  Group | 13.485 (6.267 - 20.704)  -.006 (-.037 - .025)  .014 (-.035 - .062)  -.068 (-.278 - .141)  -.066 (-.128 - -.003)  .254 (.092 - .415)  .691 (.047 - 1.336)  -.177 (-.656 - .302)  -.751 (-1.810 - .307) | 3.624  0.16  .024  .105  .031  .081  .323  .240  .531 | -  -.036  .076  -.083  -.249  .361  .280  -.073  -.150 | <.001***  .724  .579  .517  .040*  .003**  .036*  .464  .161 |

Note: N=85. R^2^= .078 for Step 1 (*p*=.161). ∆R^2^= .198 for Step 2 (*p*< .001***). ∆R^2^= .019 for Step 3 (*p*< .001***).

**Video B**

Step 1 of the regression model was statistically significant, R^2^= .212, *F*(4, 80)= 5.372, *p*< .001, adj. R^2^= .172; however, receptive vocabulary was the only control variable that individually predicted narrative coherence scores for Video B (β= .301, *p*= .024)*.*

Step 2 of the model was also statistically significant, R^2^= .316, *F*(7, 77)= 5.076, *p*< .001, adj. R^2^= .254, explaining an additional 10% of unique variance once the control variables had been accounted for (∆R^2^=.104). In this step, Mentalising was the only significant predictor of narrative coherence scores for Video B (β= .345, *p*= .003), with receptive vocabulary becoming non-significant (β= .192, *p*= .147). EF scores were not significant (EF1: β= .116, *p=* .349; EF2: β= -.066, *p=* .498).

In the final model (Step 3), the inclusion of Group significantly predicted 4% of additional variance overall, ∆R^2^= .039, *F*(8, 76)= 5.234, *p*< .001 (Group: β= -.219, *p=* .034), with Mentalising remaining significant as a predictor variable (β= .311, *p*= .006). See **Table 5**.

**Table 5. Hierarchical multiple regression predicting total narrative coherence scores for Video A from control variables, Mentalising and EF scores, and diagnostic group (final model).**

| **Variables** | **B (95% CI)** | **SE B** | **β** | ***p*** |
| --- | --- | --- | --- | --- |
| Constant  Age  Receptive vocabulary  Expressive language  Non-verbal ability  Mentalising**  EF1  EF2  Group* | 3.353 (-3.909 - 10.615)  .018 (-.013 - .049)  .044 (-.005 - .093)  -.011 (-.222 - .199)  .001 (-.062 - .064)  .230 (.067 -.392)  .104 (-.544 - .752)  -.167 (-.649 - .315)  -1.153 (-2.218 - -.088) | 3.646  .016  .025  .106  .032  .082  .325  .242  .535 | -  .113  .230  -.013  .004  .311  .040  -.065  -.219 | .361  .248  .080  .915  .971  .006**  .750  .492  .034* |

Note: N=85. R^2^= .212 for Step 1 (*p<* .001***). ∆R^2^= .104 for Step 2 (*p*< .001***). ∆R^2^= .039 for Step 3 (*p*< .001***).
